# Improved maternity care if midwives learn to perform ultrasound: a qualitative study of Rwandan midwives’ experiences and views of obstetric ultrasound

**DOI:** 10.1080/16549716.2017.1350451

**Published:** 2017-08-02

**Authors:** Sophia Holmlund, Joseph Ntaganira, Kristina Edvardsson, Pham Thi Lan, Jean Paul Semasaka Sengoma, Annika Åhman, Rhonda Small, Ingrid Mogren

**Affiliations:** ^a^ Department of Clinical Sciences, Obstetrics and Gynecology, Umeå University, Umeå, Sweden; ^b^ Judith Lumley Centre, La Trobe University, Melbourne, Australia; ^c^ School of Public Health, College of Medicine and Health Sciences, University of Rwanda, Kigali, Rwanda; ^d^ Department of Dermatology and Venereology, Hanoi Medical University, Hanoi, Vietnam

**Keywords:** Rwanda, nurse midwives, ultrasonography, obstetrics, pregnant women

## Abstract

**Background**: Obstetric ultrasound has become an indispensable part of antenatal care worldwide. Although the use of ultrasound has shown benefits in the reduction of maternal and foetal morbidity and mortality, it has also raised many ethical challenges. Because of insufficient numbers of midwives in Rwanda, uncomplicated pregnancy care is usually provided by nurses in local health centres. Obstetric ultrasound is generally performed by physicians at higher levels of healthcare, where midwives are also more likely to be employed.

**Objectives**: To explore Rwandan midwives’ experiences and views of the role of obstetric ultrasound in relation to clinical management, including ethical aspects.

**Methods**: A qualitative study design was employed. Six focus group discussions were held in 2015 with 23 midwives working in maternity care in rural and urban areas of Rwanda, as part of the CROss Country Ultrasound Study (CROCUS).

**Results**: Obstetric ultrasound was experienced as playing a very important role in clinical management of pregnant women, but participants emphasised that it should not overshadow other clinical examinations. The unequal distribution of ultrasound services throughout Rwanda was considered a challenge, and access was described as low, especially in rural areas. To increase the quality of maternity care, some advocated strongly for midwives to be trained in ultrasound and for physicians to receive additional training. In general, pregnant women were perceived both as requesting more ultrasound examinations than they received, and as not being satisfied with an antenatal consultation if ultrasound was not performed.

**Conclusions**: Obstetric ultrasound plays a significant role in maternity care in Rwanda. Increasing demand for ultrasound examinations from pregnant women needs to be balanced with medical indication and health benefits. Training of midwives to perform obstetric ultrasound and further training for physicians would help to address access to ultrasound for greater numbers of women across Rwanda.

**RESPONSIBLE EDITOR** Virgilio Mariano Salazar Torres, Karolinska Institute, Sweden

## Background

Antenatal care (ANC) plays an important role in reducing maternal and infant morbidity and mortality [1]. ANC links the pregnant woman and her family with the health system, which, in turn, increases the likelihood of assistance by a skilled birth attendant. The World Health Organization (WHO) recommends a minimum of four ANC visits. Essential elements of ANC are vaccination, screening and treatment for infections, identification of risk factors and complications during pregnancy, provision of information related to pregnancy, childbirth and the postpartum period, and assistance with family planning [1]. Maternal mortality is a global major public health problem [2]. Worldwide, haemorrhage is the leading direct cause of maternal death, followed by hypertension or pre-eclampsia, sepsis, abortion and embolism [3]. Adequate healthcare, provided by clinically trained and certified healthcare professionals such as midwives, obstetric nurses or physicians before, during and after childbirth, has been identified as the single most important factor in saving the lives of pregnant women and newborns [4].

Obstetric ultrasound has become an indispensable part of ANC around the world [5], although ultrasound imaging may not be universally available in low-resource settings [6]. Ultrasound during pregnancy is generally used to estimate gestational age, number of foetuses, placental localisation, screening of foetal malformation, foetal growth and foetal presentation [7,8]. Ultrasound may contribute to timely diagnosis and intervention that can result in the reduction of foetal morbidity and mortality [9]. In low-income countries, ultrasound during pregnancy is generally provided by obstetricians, radiologists and medical officers [10], but a lack of trained healthcare providers and a shortage of ultrasound equipment are often barriers to its utilisation [11,12]. Many pregnant women have a strong desire for ultrasound examinations to obtain information on foetal well-being and images of the foetus [13]. The ultrasound image may also result in an increased personification of the foetus and initiate the process of bonding between the expectant parents and the foetus [13]. While there is no doubt that ultrasound plays an important role in pregnancy management, concerns have been raised that the use of ultrasound contributes to an increasing medicalisation of pregnancy and childbirth [14]. Furthermore, ultrasound has led to a greater focus on the foetus, which potentially gives rise to a maternal–foetal conflict of health interests [14,15]. The misuse of ultrasound for sex identification and subsequent sex selection are also important issues [16].

### The Rwandan setting

Rwanda has made rapid progress in economic and social development since the 1994 genocide, when approximately one million people were killed and much of the socio-economic fabric of Rwanda, as well as many health facilities, were destroyed [17]. Rwanda is one of the nine countries that reached the Millennium Development Goal (MDG5) that called for a reduction of 75% in the maternal mortality ratio (MMR) from 1300 per 100,000 live births in 1990 to 290 per 100,000 live births in 2015 [18,19]. The Rwandan Demographic and Health Survey 2015 reports an MMR rate of 210 per 100,000 live births [20]. The main reasons for the improvement in MMR in Rwanda are an increasing number of pregnant women giving birth in health facilities attended by skilled healthcare professionals, the introduction of a maternal death audit [21] and the installation of community health workers [22]. From 1990 to 2015, skilled birth attendance increased from 17% to 83% [19].

A partnership of government programmes, a community-based health insurance scheme (Mutuelle de Santé) and a performance-based financing system, has been organised as a means of reaching the goal of becoming a middle-income country by 2020 [23,24]. Rwanda is still facing a severe shortage of health professionals, especially midwives [25]. Before 1997, there was no formal midwifery education in Rwanda. Since then, various reforms and initiatives have been undertaken to increase the number of midwives and the quality of care provided by midwives [26]. Currently, midwives work mainly at district hospitals or at higher healthcare levels [27]. In 2015, there were 910 midwives, 8751 nurses and 742 physicians registered in Rwanda [25]. According to the WHO, to ensure that 95% of all deliveries are attended by a skilled birth attendant, to reduce maternal and newborn mortality, a minimum of six health workers with midwifery skills is required per 1000 births [28]. In Rwanda, there are approximately 300,000 births per year [25]. Because of insufficient midwives, pregnancy surveillance and delivery care are mostly provided by nurses at health centres, with referrals to higher levels of healthcare in case of complications [29]. There are three educational levels for nurses and midwives: A0, with a bachelor’s degree after 4 years of training at university level; A1, with a diploma after completing 3 years at an institute of higher education; and A2, with lower level training within the secondary school system [30]. None of these educations includes ultrasound training [31,32]. The educational track for A2 nurses was discontinued by the Ministry of Health in 2006 in an attempt to upgrade competence within the health sector. A2 nurses are, however, still very common [30,33].

Despite the lack of trained healthcare providers, Rwanda has adopted the WHO’s model of four ANC visits for women without risk factors or pregnancy-related complications [34]. Currently, 99% of pregnant women in Rwanda attend at least one ANC visit and 44% receive at least the recommended four ANC visits [20]. In one of the previous studies undertaken by the CROCUS research group, it was identified that no physicians were permanently posted at health centres and physicians managing pregnant women in district hospitals were mainly general practitioners. Obstetric ultrasound scans are generally provided at district and referral hospitals, but also at private clinics [35]. Only physicians are formally approved to perform ultrasound examinations and the majority of physicians have no or limited formal training in ultrasound surveillance during pregnancy [35]. The Ministry of Health in Rwanda has issued guidelines for clinical management in gynaecology and obstetrics [36], but specific guidelines for the use of obstetric ultrasound are limited.

### Rationale of the study

Physicians are the health professionals who mainly perform obstetric ultrasound examinations in Rwanda, although they do not provide the majority of maternity care. To be able to make further improvements in the access and quality of obstetric ultrasound in Rwanda, it is also important to take into account the perspectives of midwives on the use of obstetric ultrasound. This is currently a knowledge gap in the literature; to fill this gap, the aim of this study was to explore Rwandan midwives’ experiences and views of the role of obstetric ultrasound in relation to clinical management, including ethical aspects.

## Methods

### Study design

A qualitative study design was employed. Data were collected through focus group discussions (FGDs) with midwives and analysed using content analysis [37]. This study is part of the CROss Country Ultrasound Study (CROCUS), an international study that aims to explore midwives’ and obstetricians’ experiences and views of the use of ultrasound in pregnancy management in low-, middle- and high-income countries. The countries participating in CROCUS are Rwanda, Tanzania, Vietnam, Australia, Norway and Sweden.

### Recruitment of participants

Three district hospitals, two university teaching hospitals and one private hospital in rural and urban areas of Kigali and in the Southern province of Rwanda were selected for recruitment. The number of births at the selected hospitals ranged between 1400 and 4500 annually. The hospitals were purposively selected to include participants at all healthcare levels that provide obstetric ultrasound in urban and rural areas, to gain a broad range of experiences. The recruitment was organised by the two local researchers, JN and JPS. The Ministry of Health approved the study and the heads of the selected health facilities assisted with the recruitment of participants working within maternity care. Participants of varying age and with various lengths of work experience were sought. Because of the high workload at the study sites, all available midwives at the time of each FGD were invited to participate, regardless of their background. The size of the focus groups varied from two to six participants depending on the availability of midwives at each selected health facility.

### Participants’ characteristics

Twenty-three midwives participated in the study, all females. The mean age was 32.6 years (range 25–47 years). The mean length of work experience in maternity care was 6.5 years (range 1 month to 19 years). Two participants reported special training in ultrasound. Participant characteristics are presented in Table 1.

**Table 1.** Characteristics of midwives (*n* = 23) participating in focus group discussions (FGDs).

FGD	Workplace: rural/urban hospital	*n*	Mean age (years)	Work experience as a midwife (years) mean (range)
A	Urban	3	32.7	2.7 (2–4)
B	Rural	6	34.8	12.5 (7–19)
C	Rural	2	30.5	6.5 (3–10)
D	Urban	5	30.6	3.2 (2–6)
E	Urban	3	37.3	7.5 (2.5–15)
F	Urban	4	29.5	3.9 (0.1–7)
All		23	32.6	6.5 (0.1–19)

### Data collection procedures

Data collection was undertaken during 1 week in January 2015. Before the start of each FGD, all participants were provided with written information about the study, a consent form to sign and a brief questionnaire on their background characteristics. The FGDs were held in Kinyarwanda. To make sure that all topics of interest were covered in each FGD, an interview guide, previously developed by the CROCUS team, was used (Table 2). The same interview guide has been used across all six countries participating in CROCUS. The topics were not discussed in a predefined order, to allow participants to talk freely and at length. The FGDs were conducted by one member of the research team (JPS), and two other members (KE and AÅ) attended as observers. Notes were taken on informal communication and any disrupting factors, for example interruption because of a medical emergency at the clinic. The FGDs were digitally recorded and lasted for 25–45 min (mean of 35 min). Data saturation, i.e. where no new information was forthcoming [38], was assessed to have been reached after six FGDs.

**Table 2.** Key domains in the CROss Country Ultrasound Study (CROCUS) interview guide.

The midwives’ experiences and views of:
1. The role of obstetric ultrasound for clinical management of complicated pregnancy.
2. The role of obstetric ultrasound in comparison to other surveillance methods during complicated pregnancy.
3. Clinical situations where the interests of maternal and foetal health conflict.
4. Whether the woman may be considered to act as an instrument for foetal treatment.
5. If/when the foetus can be regarded as a person.
6. Situations where the foetus has been regarded as a patient with his/her own interests.
7. Their professional role in relation to other occupational groups working with obstetric ultrasound examinations or the outcomes of these examinations.
8. Their perception of the community’s views of obstetric ultrasound.
9. Other issues in relation to ethical aspects of the use of obstetric ultrasound.

### Data analysis

All recordings were transcribed verbatim and translated from Kinyarwanda to English by an external person. The moderator (JPS) control-read the transcriptions while simultaneously listening to the recorded FGD. To check the consistency of the translation, another person outside the research team back-translated parts of the English version to Kinyarwanda. Some specific words were found to differ, but the sense of the whole was the same. Data were analysed using qualitative content analysis, inspired by Graneheim and Lundman [37]. The transcriptions were read several times by two of the authors (SH and IM) to gain a sense of the whole. First, meaning units were identified, condensed and coded by SH. Secondly, all coded data were reviewed by IM and some additional codes were noted. Thirdly, SH and IM discussed and sorted the codes into content areas, based on their similarities and differences. Thereafter, the content areas were categorised into preliminary categories and subcategories. SH and IM discussed the findings of the primary analysis, and an overall theme, four categories and their 10 subcategories were developed during this process. The analysis was an iterative process, moving back and forth between text, codes, subcategories and categories [37]. Examples of text, codes and categories generated from content analysis of FGDs are presented in Table 3. In cases of uncertainty, the recordings were listened to again, and the transcribed text was reanalysed to clarify the participants’ statements. Later during analysis, all authors reviewed the preliminary results and discussed the findings until consensus was reached.

**Table 3.** Examples of text, codes and categories generates from content analysis of focus group discussions.

Text	Codes	Categories
It would be good if there was a nurse or a midwife trained in ultrasound at the health centre level	Ultrasound training for nurses and midwives	Advocating for development of maternity care
	Good with ultrasound at health centres	
Every woman wants to use echography, and if you don’t perform it, she does not feel happy at all, even if you give her other types of care required. She feels that something important is missing	Everyone wants ultrasound	Differing views on use of obstetric ultrasound
	Women not happy at all being without ultrasound at antenatal care visits	
	Women valuing ultrasound more than other examinations	
	Other types of care required in addition to ultrasound	
	Something important is missing without ultrasound	

## Results

The overall theme ‘Balancing increasing demands and modern technology in the context of limited resources’ emerged during the analysis. The theme was created from four main categories: ‘Limited resources cause shortcomings in maternity care’, ‘Advocating for development of maternity care’, ‘Differing views on use of obstetric ultrasound’ and ‘Balancing maternal and foetal health interests’. The theme, categories and their subcategories are presented in Table 4.

**Table 4.** Theme, categories and subcategories.

Theme	Categories	Subcategories
Balancing increasing demands and modern technology in the context of limited resources	Limited resources cause shortcomings in maternity care	Challenges due to insufficient and unequal distribution of resources
		Lack of physicians means increased responsibility for midwives
	Advocating for development of maternity care	Obstetric ultrasound – only the duty of physicians’?
		Midwives arguing for change in maternity ultrasound services
	Differing views on use of obstetric ultrasound	Obstetric ultrasound not always an undisputed tool
		Pregnant women demanding ultrasound
		Religious and spiritual beliefs clashing with modern technology
	Balancing maternal and foetal health interests	Prioritising maternal health interests Enduring treatment for the sake of the foetus
		Respecting pregnant women***’***s autonomy in counselling

### Limited resources cause shortcomings in maternity care

This category includes two subcategories: ‘Challenges due to insufficient and unequal distribution of resources’ and ‘Lack of physicians means increased responsibility for midwives’. Both subcategories present aspects of a lack of resources in maternity care. The first subcategory describes differing access to ultrasound and the impact of absence of ultrasound examinations. The second subcategory presents participants’ experiences of how their work was affected by a lack of physicians.

#### Challenges due to insufficient and unequal distribution of resources

The unequal distribution of obstetric ultrasound services across the country and between different socio-economic groups was considered a challenge. It was perceived that pregnant women were commonly aware of the importance of ultrasound during pregnancy. However, access to obstetric ultrasound was often poor, particularly for women living in rural areas.

'People from remote areas know about it [ultrasound] and would want to use it, but they cannot easily come here because they live far away from the hospital.' (FGD A)

The availability of ultrasound examinations in a normal pregnancy was said to be linked to whether the pregnant woman had health insurance or could afford to pay. If a pregnancy was considered as normal, a pregnant woman would not be referred from a health centre to a hospital for an ultrasound examination. The participants’ conclusion was that many pregnant women, especially in rural areas, gave birth at facilities where the birth attendant had little information about the status of the pregnancy. Thus, lack of a general routine ultrasound could make it difficult for health professionals to manage pregnant women before or during labour as information on gestational length, number of foetuses, localisation of the placenta and foetal anatomy were not available. It was said that some hospitals therefore performed an ultrasound examination on all pregnant women to acquire additional obstetric information.

'Each woman referred [to a higher level of healthcare] for pregnancy complication, abortion, or any other pregnancy-related issue, medical examinations are systematically performed, including ultrasound.' (FGD A)

#### Lack of physicians means increased responsibility for midwives

Insufficient numbers of physicians in maternity and delivery care was depicted as a common problem. Emergency ultrasounds could sometimes not be performed, for example if the physician was busy in the operating theatre. A particularly challenging situation was described when the participants had no resources to assist pregnant women at risk, for example when an ultrasound machine was available but no one could operate it. The unavailability of physicians in emergency situations led to participants making their own decisions, which included performing ultrasound examinations, for example to assess foetal heart rate and foetal presentation, even though they had no formal ultrasound training.

'There are times when you receive a woman who needs to be checked with ultrasound but the doctor is not around. But if you were able to use it [ultrasound] you could document it and make a decision. But since we are not allowed it is not our responsibility, we do it secretly and no decision is made.' (FGD B)

### Advocating for development of maternity care

This category includes two subcategories: ‘Obstetric ultrasound – only the duty of physicians’?’ and ‘Midwives arguing for change in maternity ultrasound services’. Both subcategories include aspects of how Rwandan maternity care can be developed to increase quality of care. The first subcategory presents participants’ views of the division of work tasks between midwives and physicians and who can perform ultrasound examinations. The second subcategory describes participants’ wishes to learn to perform obstetric ultrasound examinations and the perceived need for ultrasound services at health centre level.

#### Obstetric ultrasound – only the duty of physicians’?

On admission of a pregnant woman to hospital, the initial assessment was generally performed by midwives. Evaluating the foetal heart rate was seen as one of the most essential duties. Performing ultrasound was generally considered to be the physician’s duty, even though many participants reported that they would have appreciated learning how to perform the examination.

'We perceive that it is not out our job, but our wish as midwives is to be able to perform ultrasound so that we can play a role in the mother’s care and make decisions without necessarily waiting for the availability of the doctor.' (FGD B)

Use of obstetric ultrasound differed between hospitals, from physicians performing ultrasound routinely on all pregnant women, whether during antenatal consultations or in labour, to only performing ultrasound if the midwife detected any pregnancy deviation. Midwives commonly described assisting the physician during ultrasound examinations. This situation was also seen as a learning experience, and occasionally a midwife would ask the physician to show her how to perform the ultrasound and interpret the image.

'A few things we know [use of ultrasound] is what we have learnt by just observing when the doctor is using it, he can show you how to check foetal heart rate. But we did not receive any training and we have limited skills on its use.' (FGD D)

If a midwife detected a deviation on ultrasound, the physician was informed and commonly wanted to confirm the result. Participants commented that some physicians believed that ultrasound should not be performed by midwives and therefore they did not trust the ultrasound results presented by the midwives. One participant mentioned that she only performed ultrasound when she knew that the physician was not nearby.

'Sometimes, when the doctor is in the operating theatre, you can take advantage of his absence and do an ultrasound to check the presentation of the baby, or examine a woman complaining about the lack of foetal movements.' (FGD B)

#### Midwives arguing for change in maternity ultrasound services

The participants argued for providing obstetric ultrasound services at health centres as it was seen as one way to increase the quality of maternity care. Insufficient access to ultrasound was sometimes experienced to result in a delayed referral to the hospital. The participants further suggested that the workload for the physicians at hospitals would decrease if ultrasound were available at health centres. It was reported that there were no physicians working permanently at health centres, but the participants saw the potential for midwives and nurses to be trained in ultrasound. It was apparent during the discussions that participants wanted ultrasound training, and it was seen as particularly important for midwives and nurses working at health centres in rural areas.

'We want you to use the results of your research to advocate for us and say that there are people [midwives] who take care of pregnant women and are involved in helping the doctor, especially a midwife who works in a rural area where a pregnant woman comes to give birth without having any information about that pregnancy. So we need training and ultrasound machines in order to help [pregnant] women in rural areas, especially those who are very far away and it is not easy for them to reach a district hospital where they can find doctors. So it would be good if there were a nurse or midwife trained in ultrasound use at the level of the health centre as there are no doctors at that level.' (FGD E)

The participants identified a major need for more healthcare professionals to be performing ultrasound examinations, and suggested that it could become part of midwives’ duties. The participants were also aware of midwives performing ultrasound examinations in other countries. They thought that this might be a missed opportunity, especially for a low-resource setting such as Rwanda. However, they reported that neither physicians nor midwives attended formal training in ultrasound. Physicians learnt from each other in practice, but participants believed that there was also a need for physicians to undergo formal ultrasound training.

'Maybe we cannot now have gynaecologists at all hospitals, but training for doctors is needed ....' (FGD B)

### Differing views on use of obstetric ultrasound

This category includes three subcategories: ‘Obstetric ultrasound not always an undisputed tool’, ‘Pregnant women demanding ultrasound’ and ‘Religious and spiritual beliefs clashing with modern technology’. All subcategories include participants’ views on society’s impact on ultrasound use. The first subcategory describes a major impact on quality of maternity care when ultrasound was introduced but also raises concerns about the risk of negative side-effects of its implementation. The second subcategory highlights the participants’ perception of pregnant women’s desire for ultrasound examinations. The last subcategory reports aspects of how religion may affect the view of the foetus and how it might influence ultrasound examinations.

#### Obstetric ultrasound not always an undisputed tool

Over the past decade in Rwanda, participants had experienced obstetric ultrasound as playing a very important role in decreasing risks in pregnancy and improving pregnancy outcomes. Since ultrasound had been introduced as a method of surveillance during pregnancy, it was described as being much more common that severe foetal conditions and deviations were detected before birth. The number of foetal deaths had, according to the participants, therefore been reduced.

'Ultrasound has played a great role in the reduction of babies’ deaths.' (FGD D)

Although ultrasound was considered very useful, participants noted that it should not overshadow other clinical examinations in ANC. Concerns were raised over whether ultrasound could be harmful for the foetus, and some participants expressed a fear that there might be negative long-term effects of ultrasound that were unknown.

'For me, we may be acting against the rights of the baby if ultrasound has got side-effects harmful to the child; but as long as ultrasound is harmless, I do not see any issue in using it.' (FGD B)

#### Pregnant women demanding ultrasound

Pregnant women in general were perceived to want more ultrasound examinations than they received. Participants described many pregnant women trying to find ways to have an ultrasound examination, for example by reporting that they could not feel foetal movements.

'She [the pregnant woman] asks you ‘How can I proceed to get checked with ultrasound? … What can we do if our health centre does not give us a transfer? We need it’. You can realise that they [pregnant women] are not happy about the fact that the [health] centre does not give them a transfer for ultrasound.' (FGD D)

Information on foetal sex and foetal well-being, and curiosity about the unborn child were reported to be the major reasons for pregnant women wanting to have an ultrasound. Pregnant women were often informed about the sex of the foetus at the ultrasound examination as it was said to be important for their preparations before birth. However, participants mentioned that it could be unsettling for the mother to receive information about a sex that she did not wish for. Some pregnant women did not trust the information regarding the sex of the baby, and therefore had several ultrasounds with different physicians for confirmation.

'What often happens in ultrasound is you tell the woman the sex of the child she did not want. I think that this can be disturbing psychologically for the mother and, in the end, it can be harmful to the child.' (FGD B)

Affluent women with private health insurance were commonly reported as receiving at least three ultrasounds during pregnancy. Even though some pregnant women had several ultrasound examinations, they were sometimes still not satisfied. Participants even expressed that some pregnant women overused ultrasound when it was available but not medically indicated. Participants also mentioned that some pregnant women believed that they avoided pregnancy risks if they were examined by ultrasound, or they considered ultrasound examinations as some kind of treatment.

'Some women take it as a routine exam, she thinks that she has to have it every time that she comes, and it is here that we as midwives have to play our role of showing them the importance of ultrasound and make them understand that having an ultrasound examination every day is not adding any value.' (FGD E)

Participants experienced that pregnant women relied more on ultrasound than on other examinations during pregnancy. When the foetal heartbeat was assessed with a Pinard horn, i.e. a trumpet-shaped horn to monitor the foetal heart rate [39], a common experience was that pregnant women did not trust this examination. Furthermore, pregnant women were said not to be satisfied with an antenatal consultation if ultrasound was not performed.

'Psychologically she feels well and knows that her child is healthy [when examined by ultrasound]. However, when we use the foetoscope [Pinard horn], they do not trust it and question its result .... They trust results provided by ultrasound.' (FGD C)

#### Religious and spiritual beliefs clashing with modern technology

Pregnant women in general were considered to be very cautious about their pregnancies. Even though most people in the society did not view the foetus as a child, it was reported that many pregnant women did consider the foetus as a human being, especially when it was a desired pregnancy.

'From my own experience, most women consider a foetus as a human being. In many cases, a woman gets pregnant with a wish to have a child, and if she is lucky and does not abort the pregnancy, she considers the foetus as her child.' (FGD B)

Pregnant women were said to become confused when a foetal malformation was detected at ultrasound examination. Low educational level was presumed to be the reason that some pregnant women had little understanding of the cause of malformations. Some pregnant women did not accept terminating the pregnancy even if recommended when a severe foetal malformation was diagnosed by a physician. Religious beliefs were understood to affect the view of the foetus and induced abortion. Some participants were upset about the Church providing pregnant women with false prophecy on the sex of the foetus and foetal malformation. Pregnant women sometimes sought more ultrasound examinations to confirm the sex of the foetus because the Church had told them that it was the opposite sex. Furthermore, some pregnant women believed in witchcraft and therefore they wanted an ultrasound examination to confirm that it was a human being inside the womb.

'She [pregnant woman] may be worried about witchcraft, and suspects that she is carrying an animal.' (FGD F)

### Balancing maternal and foetal health interests

This category includes three subcategories: ‘Prioritising maternal health interests’, ‘Enduring treatment for the sake of the foetus’ and ‘Respecting pregnant women’s autonomy in counselling’. Common among these subcategories are aspects of standpoints regarding pregnancy complications. The first category presents experiences of how maternal health was mostly prioritised over foetal life. The second subcategory describes attitudes towards treatment in pregnancy aiming primarily to benefit the foetus. The third subcategory describes the experiences of counselling pregnant women in severe obstetric situations and the collaboration with physicians in these situations.

#### Prioritising maternal health interests

Most participants had faced situations when decisions had been made to save either the pregnant woman or the foetus. In severe medical conditions, the pregnant woman’s health was mostly prioritised over that of the foetus.

'When we find that the mother is running a very high risk of dying, we make the decision to terminate the pregnancy.' (FGD F)

'They [physicians] may want to save the baby or the mother depending on their chances of survival.' (FGD A)

#### Enduring treatment for the sake of the foetus

Ultrasound examinations were perceived as important for making decisions regarding whether treatment of pregnancy complications was possible. Several examples were reported where the foetus was receiving treatment by treating the pregnant woman. A common case was to give corticosteroids to enhance foetal lung maturation in the case of threatening premature delivery. In general, there were no concerns about the side-effects of drugs given to the pregnant woman. It was explained that there was a need to balance potentially harmful maternal effects against the positive foetal effects expected when treatment was given. The participants thought that most pregnant women were positive about receiving treatment for the sake of the foetus.

'When she [the pregnant woman] understands that you are going to do something to help her baby, she does not refuse, she bears with it.' (FGD F)

The participants reported that they had never experienced a foetus being given treatment directly through the pregnant woman’s uterus. A few participants were aware of treatment through foetal blood transfusion, but it was not known to have occurred in Rwanda.

#### Respecting pregnant women’s autonomy in counselling

Midwives involved in maternity care at hospitals provided information to pregnant women about ultrasound examination and its advantages.

'… the role that we play is to counsel all women who come to us in ANC. We have to give them advice, that is our responsibility, we talk to them about the ultrasound and its importance ….' (FGD E)

It was recognised that most pregnant women wanted an ultrasound examination, and the participants saw this as an opportunity to explain the medical reasons for it. Pregnant women were sometimes encouraged to request the physician’s services for an ultrasound examination since the midwives thought that it was the pregnant woman’s right to receive it. Participants sometimes needed to explain the results of the examination to the patient when the physician did not have time to explain all the details.

'Sometimes, the doctor does not have enough time to explain all the details revealed by ultrasound and as we meet the mother many times, we explain all the details about things checked to her.' (FGD B)

Since the midwives assisted the physician at all times when ultrasound was performed, this meant that they were present when deviations were detected. The physician mainly informed women about the results of the ultrasound examination but the participants reported that they contributed to the discussion of termination of the pregnancy when a malformation was diagnosed. Involving family members was considered important, especially when making difficult decisions, such as termination of the pregnancy. The participants felt that they had an important role to play into supporting and counselling pregnant women; however, it could also at times be very demanding and difficult. Providing adequate information to pregnant women was considered very important. No intervention could be performed without the consent of the pregnant woman, and if a pregnant woman declined to terminate a pregnancy, her choice was respected.

## Discussion

The present study investigated Rwandan midwives’ experiences and views of the use of obstetric ultrasound. Ultrasound was considered a crucial tool in maternity care even though access to and utilisation of ultrasound varied greatly across Rwanda. Participants thought that physicians managing pregnant women need additional training in performing obstetric ultrasound, and they suggested that ultrasound training also for midwives to improve access to ultrasound for pregnant women. Pregnant women were seen not only as valuing obstetric ultrasound but also as generally wanting more obstetric ultrasound examinations than they received.

### Obstetric ultrasound in low-resource settings

A review of the use of ultrasound in low-income countries concludes that obstetric ultrasound is a valuable tool in these settings [40]. Although Rwanda is a geographically small country there seem to be large differences in access to obstetric ultrasound between urban and rural areas. Results from other low-income countries in Africa, South America and Asia show that insufficient training for healthcare providers is the primary barrier to regular use of obstetric ultrasound [12]. Most participants thought that there was a need for formal training for physicians, but also introduction of ultrasound training for midwives, to help to decrease the burden of work in hospitals and to improve access to ultrasound at healthcare centres, particularly in rural areas. To achieve a high quality of ultrasound examinations, the WHO strongly recommends that appropriate curricula be adopted for general, advanced and specialised training of physicians and other healthcare professionals who perform diagnostic ultrasound [41].

### Midwives as potential operators of obstetric ultrasound

The participants reported that in clinical practice, obstetric ultrasound was the physician’s duty. Some midwives performed ultrasound examinations, but mainly in urgent clinical situations that required an ultrasound evaluation when physicians were not available. The hospital-based participants pointed out that there was an explicit shortage of physicians in maternity care and they suggested that performing ultrasound could be part of the midwife’s work as well as the physician’s. It can be both feasible and practical in a low-resource setting to train midwives in order to increase the use of ultrasound in ANC [11]. It has been argued that training in ultrasound should be directed towards midwives as well as physicians, as midwives provide most of the maternity care [42]. The effectiveness of training midwives in obstetric ultrasound has been evaluated as successful in several African countries [43–45]. In an intervention study conducted in Zambia, 17% of the scans performed by midwives resulted in a change in clinical decision making [43]. It has also been shown that obstetric ultrasound used as a screening and diagnostic tool by midwives with selected risk groups increases the chance of establishing diagnoses compared with clinical examination only [44].

### Organisation of obstetric ultrasound

In our study, it was suggested that the health system would be likely to benefit if obstetric ultrasound was performed at the level of health centres. Other studies have shown that introducing obstetric ultrasound at the lowest level of the healthcare system in a low-resource setting increases ANC attendance and referral of high-risk pregnancies, and can motivate women to give birth at a health facility [46,47]. The participants also wished to decrease the gap between different socio-economic groups in relation to access to obstetric ultrasound. Because of Rwanda’s community-based health insurance system, the country has demonstrated substantial improvements in maternal health over the past decade [48]. However, the participants reported that community health insurance did not cover the costs of obstetric ultrasound if the pregnancy was presumed to be normal. To further reduce maternal and foetal morbidity and mortality in Rwanda, the benefits of a routine ultrasound examination during the second trimester of pregnancy [49–51], covered by community health insurance, should be evaluated.

### Obstetric ultrasound for medical reasons in contrast to ultrasound on request

According to participants, many pregnant women were strongly motivated to have an ultrasound examination. Other studies also show that ultrasound is very attractive to pregnant women and their families [52,53], but at the same time pregnant women often overestimate the diagnostic capacity and the therapeutic possibilities that may follow an ultrasound examination [53]. The attractiveness of ultrasound for pregnant women may be due to the early visual confirmation of pregnancy, a ‘contact’ with the unborn child and reassurance about foetal well-being [52]. It was also reported in our study that pregnant women did not always trust other clinical examinations used for pregnancy surveillance, while trust in ultrasound was described as high.

Even though ultrasound was considered very important in maternity care, potential risks associated with overuse were raised. Furthermore, performing obstetric ultrasound at every antenatal visit was viewed as not adding any valuable information in normal pregnancies. These opinions are compatible with the WHO recommendations, that providers of ultrasound need to take great responsibility in risk–benefit assessments and advise that ultrasound should be used according to recognised guidelines at the lowest necessary exposure [54].

### Methodological consideration

A strength of this study was purposive sampling of health facilities including both rural and urban areas, and health facilities of different sizes. All midwives available on the day of data collection participated in the study. A possible limitation of this study is the size of the focus groups, which varied from two to six participants. The ideal size of an FDG has been suggested to be five to eight participants [55]. The disadvantage of a small focus group is that it may limit the variety of experiences shared and the overall exchange among the participants. On the other hand, small focus groups are preferable when participants have vast experiences of the topic under study [55]. The aim had been to include more than two participants, but because of high workload at some study sites, this was not always possible. Another issue for focus group composition might be that two of the participants had participated in ultrasound training and the other participants had not. That situation may have influenced the discussions, but not necessarily in a negative way. Our sample of participants demonstrated diversity in relation to age and work experience, which contributed to a varied discussion about different experiences of maternity care. The FGDs were held in the participants’ native language, Kinyarwanda, which enabled them to express their thoughts fully. The FGDs were transcribed and translated from Kinyarwanda to English. Translation between different languages entails a risk of losing important information and of misunderstanding the participants’ statements [56]. To decrease that risk and to increase the credibility of the study, the moderator control-read the transcriptions while listening to the recorded FGDs. In addition, an independent person back-translated parts of all the translated FGDs. A possible limitation is that the FGDs were facilitated by a member of the research team who worked as a physician, i.e. had a higher position in the hierarchy of Rwandan healthcare professionals. Because of this, the participating midwives might not have felt able to fully express their opinions. However, the material indicates otherwise, since the participants seemed to discuss issues freely, even bringing up what they did in secret behind the physicians’ backs. The interview guide used in the study has been used in other low-, middle- and high-income countries included in the larger CROCUS project, although some parts, as for example the topic on foetal treatment, were less relevant for Rwanda.

A possible limitation is that nurses working at health centres were not included in the study even though they are managing most basic obstetric care. The decision not to include health centres was made because ultrasound services are generally not provided at this level of healthcare. At higher levels of healthcare, such as the health facilities included in the study, it is mainly the midwives who manage pregnant women. Many of the participants stated that it would be a great advantage if midwives at health centres could learn to perform ultrasound. We believe that this may be the general opinion of midwives working in Rwanda, although it cannot be ruled out that midwives in other parts of the health system may have had other opinions.

To ensure transferability of the findings, data collection continued until saturation was reached, i.e. no major new information was forthcoming [38]. Keeping notes on informal communication and saving the records of the FGDs to return to if needed, increased dependability in the study. Finally, the confirmability was achieved by two of the authors with different professional backgrounds (SH and IM) discussing the analysis at every step until consensus was obtained. Two other authors (JN and JPS) are Rwandan physicians and researchers, and were well acquainted with the Rwandan healthcare system, which contributed to the interpretations of the findings.

## Conclusions

Obstetric ultrasound plays a significant role in maternity care in Rwanda. Healthcare professionals have an important role to play in providing information to pregnant women and their families to facilitate informed decision making. The increasing demand for ultrasound examinations from pregnant women needs to be balanced in relation to medical indication and benefits in order to prevent overuse of obstetric ultrasound. Unequal access to obstetric ultrasound between rural and urban areas and between different socio-economic groups was reported as a major problem. To increase access to ultrasound for all pregnant women in the country, we suggest that midwives are trained to perform basic ultrasound. Increased availability of obstetric ultrasound would improve the quality of maternity care. Additional formal training of physicians in obstetric ultrasound is also recommended in order to increase the quality of ultrasound surveillance during pregnancy to improve maternal and foetal health outcomes.

## Paper context

Obstetric ultrasound is an important part of antenatal care, but access varies greatly across Rwanda. It is mainly physicians with no formal training who perform ultrasound at district hospitals. Physicians need more training in obstetric ultrasound to provide quality maternity care. Poor access to ultrasound, especially for pregnant women in rural areas, can be improved by training midwives or nurses to perform basic ultrasound examinations at the health-centre level.

## References

[1] Lawn J, Kerber K, editors. Opportunities for Africa’s newborns: practical data, policy and programmatic support for newborn care in Africa Cape Town: WHO on behalf of the Partnership for Maternal Newborn and Child Health (PMNCH); 2006 p. 51–12.

[2] World Health Organization Maternal mortality, fact sheet no. 348. World Health Organization; 2015 Available from: http://www.who.int/mediacentre/factsheets/fs348/en/

[3] Say L, Chou D, Gemmill A, et al Global causes of maternal death: a WHO systematic analysis. Lancet Glob Health. 2014;2:e323–e333. 25103301 10.1016/S2214-109X(14)70227-X

[4] Foster-Rosales A. Maternal mortality: the eye of the storm In: Murthy P, Lanford Smith C, editors. Women’s global health and human rights. Sudbury (MA): Jones and Bartlett; 2010 p. 279–286.

[5] Gammeltoft T, Nguyen HT The commodification of obstetric ultrasound scanning in Hanoi, Viet Nam. Reprod Health Matters. 2007;15:163–171. 10.1016/S0968-8080(06)29280-2 17512387

[6] Harris RD, Marks WM Compact ultrasound for improving maternal and perinatal care in low-resource settings: review of the potential benefits, implementation challenges, and public health issues. J Ultrasound Med. 2009;28:1067–1076. 19643790 10.7863/jum.2009.28.8.1067

[7] Cargill Y, Morin L, Bly S, et al Content of a complete routine second trimester obstetrical ultrasound examination and report. J Obstet Gynaecol Can. 2009;31:272–275, 6–80. 10.1016/S1701-2163(16)34127-5 19416575

[8] Bricker L, Medley N, Pratt JJ Routine ultrasound in late pregnancy (after 24 weeks’ gestation). Cochrane Database Syst Rev. 2015Jun 29;(6):CD001451. 26121659 10.1002/14651858.CD001451.pub4 PMC7086401

[9] Wiafe YA, Odoi AT, Dassah ET The role of obstetric ultrasound in reducing maternal and perinatal mortality. Ultrasound imaging- medical applications [Internet]. InTech; 2011 [cited 2016 12 12]. Available from: http://www.intechopen.com/books/ultrasound-imaging-medical-applications/the-role-of-obstetric-ultrasound-in-reducing-maternal-and-perinatal-mortality

[10] Seffah JD, Adanu RM Obstetric ultrasonography in low-income countries. Clin Obstet Gynecol. 2009;52:250–255. 19407532 10.1097/GRF.0b013e3181a4c2d5

[11] Bentley S, Hexom B, Nelson BP Evaluation of an obstetric ultrasound curriculum for midwives in Liberia. J Ultrasound Med. 2015;34:1563–1568. 26254155 10.7863/ultra.15.14.08017

[12] Shah S, Bellows BA, Adedipe AA, et al Perceived barriers in the use of ultrasound in developing countries. Crit Ultrasound J. 2015;7:11. 10.1186/s13089-015-0028-2 PMC4485671 26123609

[13] Øyen L, Aune I Viewing the unborn child – pregnant women’s expectations, attitudes and experiences regarding fetal ultrasound examination. Sex Reprod Healthc. 2016;7:8–13. 26826039 10.1016/j.srhc.2015.10.003

[14] Zechmeister I Foetal images: the power of visual technology in antenatal care and the implications for women’s reproductive freedom. Health Care Anal. 2001;9:387–400. 11874254 10.1023/A:1013837511115

[15] Fasouliotis SJ, Schenker JG Maternal-fetal conflict. Eur J Obstet Gynecol Reprod Biol. 2000;89:101–107. 10733034 10.1016/s0301-2115(99)00166-9

[16] Thiele AT, Leier B Towards an ethical policy for the prevention of fetal sex selection in Canada. J Obstet Gynaecol Can. 2010;32:54–57. 20370982 10.1016/S1701-2163(16)34405-X

[17] Logie DE, Rowson M, Ndagije F Innovations in Rwanda’s health system: looking to the future. Lancet. 2008;372:256–261. 18619670 10.1016/S0140-6736(08)60962-9

[18] Alkema L, Chou D, Hogan D, et al Global, regional, and national levels and trends in maternal mortality between 1990 and 2015, with scenario-based projections to 2030: a systematic analysis by the UN Maternal Mortality Estimation Inter-Agency Group. Lancet. 2016;387:462–474. 26584737 10.1016/S0140-6736(15)00838-7 PMC5515236

[19] World Health Organization Trends in maternal mortality: 1990 to 2015. Geneva: World Health Organization; 2015.

[20] National Institute of Statistics of Rwanda (NISR) [Rwanda], Ministry of Health (MOH) [Rwanda], and ICF International Rwanda demographic and health survey 2014–15. Rockville: NISR, MOH, and ICF International; 2015.

[21] United Nations Millennium development goals Rwanda, final progress report. Rwanda: UNDP; 2013.

[22] Sayinzoga F, Bijlmakers L Drivers of improved health sector performance in Rwanda: a qualitative view from within. BMC Health Serv Res. 2016;16:123. 27059319 10.1186/s12913-016-1351-4 PMC4826525

[23] Republic of Rwanda Rwanda vision 2020. Kigali: Ministry of Finance and Economic Planning; 2000 Jul.

[24] Pierce H, Heaton TB, Hoffmann J Increasing maternal healthcare use in Rwanda: implications for child nutrition and survival. Soc Sci Med. 2014;107:61–67. 24607667 10.1016/j.socscimed.2014.02.007

[25] National Institute of Statistics of Rwanda (NISR) Statistical yearbook. 2016 ed. (SYB2016). Kigali: NISR; November 2016.

[26] Ministry of Health Rwanda, PMNCH, WHO, World Bank, AHPSR and participants in the Rwanda multistakeholder policy review Success factors for women’s and children’s health: Rwanda. Geneva: World Health Organization; 2015.

[27] Holmlund S. Quantitative data of health care professionals’ experiences and views on obstetric ultrasound in Rwanda. (Unpublished manuscript). Sweden: Umeå University; Forthcoming.

[28] World Health Organization 10 facts on midwifery. 2014 [cited 2017 Jun 19]. Available from: http://www.who.int/features/factfiles/midwifery/en/

[29] Uwizeye G, Mukamana D, Relf M, et al Building nursing and midwifery capacity through Rwanda’s human resources for health program. J Transcult Nurs. 2017 May 3:104365961770543. 10.1177/1043659617705436 28826335

[30] Gea U Building nursing and midwifery capacity through Rwanda’s human resources for health program. J Transcult Nurs. 2017. 10.1177/1043659617705436 28826335

[31] International Confederation of Midwives International Confederation of Midwives. Rwanda; [cited 2017 6 02]. Available from: http://internationalmidwives.org/our-members/?aid=121

[32] University of Rwanda College of Medicine and Health Sciences School of Nursing and Midwifery. [cited 2017 6 05]. Available from: http://www.cmhs.ur.ac.rw/home/

[33] Ministry of Health, Rwanda. Human resources for health program Health education. 2017 [cited 2017 Jun 18]. Available from: http://www.hrhconsortium.moh.gov.rw/about-rwanda/health-education/

[34] World Health Organization Antenatal care in developing countries. Promises, achievements and missed opportunities: an analysis of trends, levels and differentials, 1990–2001. Geneva: WHO & UNICEF; 2003.

[35] Edvardsson K, Ntaganira J, Ahman A, et al Physicians’ experiences and views on the role of obstetric ultrasound in rural and urban Rwanda: a qualitative study. Trop Med Int Health. 2016 Jul;21(7):895–906. 10.1111/tmi.12718 27125579

[36] Ministry of Health Rwanda Gynecology and obstetrics, clinical protocols & treatment guidelines. Kigali, Rwanda; 2012 [cited 2017 Jun 15]. Available from: http://www.moh.gov.rw/fileadmin/templates/Clinical/OBS_Gyn_last-version.pdf

[37] Graneheim UH, Lundman B Qualitative content analysis in nursing research: concepts, procedures and measures to achieve trustworthiness. Nurse Educ Today. 2004;24:105–112. 14769454 10.1016/j.nedt.2003.10.001

[38] Fusch PI, Ness LR Are we there yet? Data saturation in qualitative research. Qual Rep. 2015;20:1408–1416.

[39] Byaruhanga R, Bassani DG, Jagau A, et al Use of wind-up fetal Doppler versus Pinard for fetal heart rate intermittent monitoring in labour: a randomised clinical trial. BMJ Open. 2015;5:1. 10.1136/bmjopen-2014-006867 PMC4316429 25636792

[40] Sippel S, Muruganandan K, Levine A, et al Review article: use of ultrasound in the developing world. Int J Emerg Med. 2011;4:1–11. 22152055 10.1186/1865-1380-4-72 PMC3285529

[41] World Health Organization Training in diagnostic ultrasound: essentials, principles and standards: report of a WHO study group. Geneva: World Health Organization; 1998. 9659004

[42] Kongnyuy EJ, van den Broek N The use of ultrasonography in obstetrics in developing countries. Trop Doct. 2007;37:70–72. 17540081 10.1177/004947550703700203

[43] Kimberly HH, Murray A, Mennicke M, et al Focused maternal ultrasound by midwives in rural Zambia. Ultrasound Med Biol. 2010;36:1267–1272. 20691916 10.1016/j.ultrasmedbio.2010.05.017

[44] Swanson JO, Kawooya MG, Swanson DL, et al The diagnostic impact of limited, screening obstetric ultrasound when performed by midwives in rural Uganda. J Perinatol. 2014;34:508–512. 24699218 10.1038/jp.2014.54

[45] Greenwold N, Wallace S, Prost A, et al Implementing an obstetric ultrasound training program in rural Africa. Int J Gynaecol Obstet. 2014;124:274–277.. 24373707 10.1016/j.ijgo.2013.09.018

[46] Mbuyita S, Tillya R, Godfrey R, et al Effects of introducing routinely ultrasound scanning during ante natal care (ANC) clinics on number of visits of ANC and facility delivery: a cohort study. Arch Public Health. 2015;73:36. 26347809 10.1186/s13690-015-0086-8 PMC4561474

[47] Kawooya MG, Nathan RO, Swanson J, et al Impact of introducing routine antenatal ultrasound services on reproductive health indicators in Mpigi District, Central Uganda. Ultrasound Q. 2015;31:285–289.. 26656991 10.1097/RUQ.0000000000000142

[48] Bucagu M, Kagubare JM, Basinga P, et al Impact of health systems strengthening on coverage of maternal health services in Rwanda, 2000–2010: a systematic review. Reprod Health Matters. 2012;20:50–61.. 10.1016/S0968-8080(12)39611-0 22789082

[49] Akinmoladun J, Ogbole G, Lawal T, et al Routine prenatal ultrasound anomaly screening program in a Nigerian university hospital: redefining obstetrics practice in a developing African country. Niger Med J. 2015;56:263–267. 26759511 10.4103/0300-1652.169705 PMC4697214

[50] McClure EM, Nathan RO, Saleem S, et al First look: a cluster-randomized trial of ultrasound to improve pregnancy outcomes in low income country settings. BMC Pregnancy Childbirth. 2014;14:73. 24533878 10.1186/1471-2393-14-73 PMC3996090

[51] Stanton K, Mwanri L Global maternal and child health outcomes: the role of obstetric ultrasound in low resource settings. World J Prev Med. 2013;1:22–29.

[52] Garcia J, Bricker L, Henderson J, et al Women’s views of pregnancy ultrasound: a systematic review. Birth. 2002;29:225–250. 12431263 10.1046/j.1523-536x.2002.00198.x

[53] Tautz S, Jahn A, Molokomme I, et al Between fear and relief: how rural pregnant women experience foetal ultrasound in a Botswana district hospital. Soc Sci Med. 2000;50:689–701. 10658849 10.1016/s0277-9536(99)00321-4

[54] World Health Organization Manual of diagnostic ultrasound. Geneva: World Health Organization; 2013.

[55] Krueger RA, Casey MA Focus groups: a practical guide for applied research. 5th ed. New Delhi: SAGE Publications Asia-Pacific Pte.Ltd; 2015.

[56] van Nes F, Abma T, Jonsson H, et al Language differences in qualitative research: is meaning lost in translation? Eur J Ageing. 2010;7:313–316. 21212820 10.1007/s10433-010-0168-y PMC2995873

